# Medical device regulation and dialysis practice—impact on patients, doctors and manufacturers

**DOI:** 10.1093/ndt/gfaf269

**Published:** 2025-12-29

**Authors:** Björn Meijers, Rukshana Shroff, Yuri Battaglia, Rumeyza Kazancioglu, Gaetano Alfano, Casper Franssen, Manfred Hecking, Valerie Luyckx, Christian Combe

**Affiliations:** Laboratory of Nephrology and Renal Transplantation, Department of Microbiology, Immunology and Transplantation, KU Leuven, Belgium; Department of Internal Medicine, Nephrology Unit, UZ Leuven, Belgium; EuDial Working Group, European Renal Association; Paediatric Nephrology Unit, University College London Great Ormond Street Hospital and Institute of Child Health, London, UK; University of Verona, Department of Medicine, Verona, Italy; Pederzoli Hospital, Nephrology and Dialysis Unit, Peschiera del Garda, Italy; Division of Nephrology School of Medicine, Bezmialem Vakif University, Istanbul, Türkiye; Nephrology Dialysis and Kidney Transplant Unit, Azienda Ospedaliero Universitaria di Modena, Modena, Italy; Department of Internal Medicine, Division of Nephrology, University Medical Center Groningen, University of Groningen, Groningen, The Netherlands; Medical University of Vienna, Department of Internal Medicine III, Clinical Division of Nephrology & Dialysis, Vienna, Austria; University Children’s Hospital, University of Zurich, Zurich, Switzerland; Department of Public and Global Health, Epidemiology, Biostatistics and Prevention Institute, University of Zurich, Zurich, Switzerland; Renal Division, Brigham and Women’s Hospital, Harvard Medical School, Boston, MA, USA; Department of Paediatrics and Child Health, University of Cape Town, Cape Town, South Africa; President European Kidney Health Alliance, Brussels, Belgium; Univ. Bordeaux, INSERM, Bordeaux Public Health, U1219, CIC 1401, Bordeaux, France; Hospices Civils de Lyon, Lyon, France; Centre Hospitalier d’Ardèche Méridionale, Aubenas, France

**Keywords:** chronic hemodialysis, dialysis, guidelines, haemodiafiltration, haemodialysis

## Abstract

The Medical Device Regulation (MDR) was adopted in 2017 to replace the Medical Device Directive. Key changes included more rigorous clinical evidence requirements, increased scrutiny of notified bodies and improved traceability of medical devices, with the overarching aim to improve their safety and quality. For chronic haemodialysis, the impact of the MDR on devices has been substantial, resulting in niche devices being no longer available, and critical shortages, especially in paediatric nephrology. The EuDial board discussed these developments and concluded that the MDR has had a clear negative impact on innovation. In conjunction with other emerging economic macro-trends, this development heightens the potential for disruptions within critical supply chains. We offer actionable recommendations to optimize the benefits of the MDR and to minimize unintended consequences.

## INTRODUCTION

The European Union (EU), over the years of its existence, has developed and adopted several guidelines for regulating medical devices. Initially, there were three directives, i.e. the Active Implantable Devices Directive (AIMDD, 1990), the Medical Devices Directive (MDD,1993) and the In Vitro Diagnostic Directive (IVDD, 1998). In 2007, amendments to the MDD introduced more explicit requirements for clinical evidence, and recognized software as a medical device.

The transition from the MDD to the Medical Devices Regulation (MDR) marked a significant overhaul. The MDR (Regulation 2017/745) was adopted to replace AIMDD and MDD with the aim to modernize and harmonize medical device regulations across EU member states. This shift, which became fully applicable on 26 May 2021, specifically aimed to enhance patient safety by imposing stricter requirements across the entire lifecycle of a medical device, from design and manufacturing to post-market surveillance.

Key changes included more rigorous clinical evidence requirements, increased scrutiny of notified bodies and improved traceability of devices. For manufacturers, these changes have necessitated a comprehensive re-evaluation of their quality management systems, technical documentation and market access strategies, ushering in a new era of compliance and accountability in the medical device industry.

In 2025, the European Commission held public consultations on the MDR and IVDR (In Vitro Diagnostic regulation), evaluating, among other things, how effective, efficient and proportionate the two regulations were in achieving robust, transparent, predictable and sustainable regulatory frameworks, ensuring a high level of safety and healthcare benefits.

Dialysis is a life-saving therapy for hundreds of thousands of individuals in Europe [1]. It relies heavily on medical devices, including dialysis filters, dialysis monitors, needles, tubing for extracorporeal circulation, dialysate concentrates and more. Therefore, the European Dialysis (EuDial) [2] Working Group of the European Renal Association (ERA) took responsibility to contribute to these public consultations, both by providing input to the Biomedical Alliance and by responding directly to specific consultations under the authority of the governing bodies of the ERA.

Issues raised during these consultations were the reduced availability of specific dialysis devices, particularly for paediatric patients, leading to critical gaps in the provision of suitable treatments. Additional concerns include deincentivizing innovation and reduced access to niche products and devices. The goal of the current manuscript is to widen these discussions, bringing them to the nephrology community at large. We describe the current availability of different types of biomedical devices for dialysis, highlight trends and assess the impact of the MDR on the innovation and manufacturing landscape. We also provide actionable recommendations to optimize benefits while minimizing unintended consequences of the MDR.

## EUROPE AS A PIONEER IN DIALYSIS

Key point:

- Europe is a leader in the innovation and development of dialysis technology, from early discoveries to modern technologies and landmark clinical trials.

Europe has made significant contributions towards the development of dialysis as a life-saving therapy. Our understanding of how kidney failure leads to the syndrome of uraemia has been shaped to a large extent by 19th century European scientists, mainly from France, Germany and the UK [3]. The first, albeit unsuccessful, human trial of extracorporeal treatment for kidney failure (Germany), as well as the first successful dialysis treatment (the Netherlands) were performed in Europe [4–6].

These pioneering years advanced dialysis as a life-saving therapy, initially as a treatment for acute kidney failure. In 1962, the Seattle Northwest Kidney Centers was the first nonprofit organization providing haemodialysis (HD) for patients with end-stage renal disease and served as a model for renal care worldwide [7]. A crucial step included the development of vascular access, with the Scribner shunt [8], and the arteriovenous fistula [9].

By the mid-1960s, a transatlantic community had brought forward new dialysis filter designs, and countries across Europe made important contributions. The concept of the capillary filter design was published as a PhD manuscript in Poland [10]. By the 1980s, over 20 dialysis filter manufacturers produced filters mostly composed of cellulose and its derivatives [11]. Starting in 1983, the production of synthetic polysulfone hollow fiber dialysers took off in Germany. Ever since, the hollow-fiber dialyser has been the global standard of care. Europe also played a significant role in the development of high-flux HD and online haemodiafiltration (HDF) [12], with several of the pivotal randomized clinical trials, e.g. the membrane permeability outcome study [13] and the Comparison of High-Dose Haemodiafiltration with High-Flux Haemodialysis (CONVINCE) study [14], performed in Europe. These studies shaped clinical practice and resulted in improved patient outcomes. International comparisons of HD quality of care indexes including mortality have always shown Europe as a reference [15].

## A MARKET PERSPECTIVE ON THE EVOLVING DIALYSIS LANDSCAPE

Key point:

- Dialysis demand in Europe is increasing due to an aging population and advances in patient-centered care.

From an economic viewpoint, the European dialysis landscape is generally considered a well-developed, mature and open market that is expected to grow significantly over time. The EU is experiencing significant demographic changes with an ageing population, driven by low birth rates and increased life expectancy. As of 1 January 2024, the EU population was estimated at 449.3 million, with 21.6% aged 65+ years [16]. Although the incidence of dialysis in elderly individuals differs between countries, ageing of the European population is expected to lead to further increases in the need for kidney replacement therapy (KRT), mainly by dialysis. This demographic shift and the growing need for KRT is illustrated by longitudinal data from the ERA registry: in 2012, the incidence of KRT was 140 per million population (pmp), increasing to 152 pmp by 2022 [17, 18]. The proportion of patients starting dialysis aged 65+ years rose from 49% in 2012 to 54% in 2022 [18]. With recent studies showing improved overall survival by HDF in comparison with high flux HD, further increases in the prevalent dialysis population are to be expected [19].

At the same time, there is a notable trend towards home dialysis, which allows patients to lead more independent lives and potentially improves survival rates compared with in-centre treatment [20, 21]. This shift is supported by technological advancements and initiatives aimed at promoting home HD and peritoneal dialysis (PD). Innovations in equipment, including portable dialysis machines and remote monitoring systems, are enhancing patient convenience and treatment outcomes. These advances are expected to drive market growth by making dialysis more accessible and efficient.

Overall, the dialysis market in Europe is poised for growth, driven by technological innovations, increasing disease prevalence, and a shift towards more convenient and patient-centred treatment options. The European HD and PD market was valued at around 26 billion EUR in 2023 and is projected to reach over 40 billion EUR by 2031, growing at a compound annual growth rate (CAGR) of 6.5% during this period [22]. Another report, using different data, estimated a slightly lower CAGR (3.8%), but similarly expects the dialysis market to grow substantially over the next 5 years.

## THE CHANGING LANDSCAPE OF THE DIALYSIS INDUSTRY

Key points:

- The European dialysis market has consolidated and globalised, reducing innovation.
- The supply of dialyser filters remains robust.
- MDR certicification of dialyser monitors is problematic.
- Paediatric nephrology faces critical device shortages.

**Figure 1:**
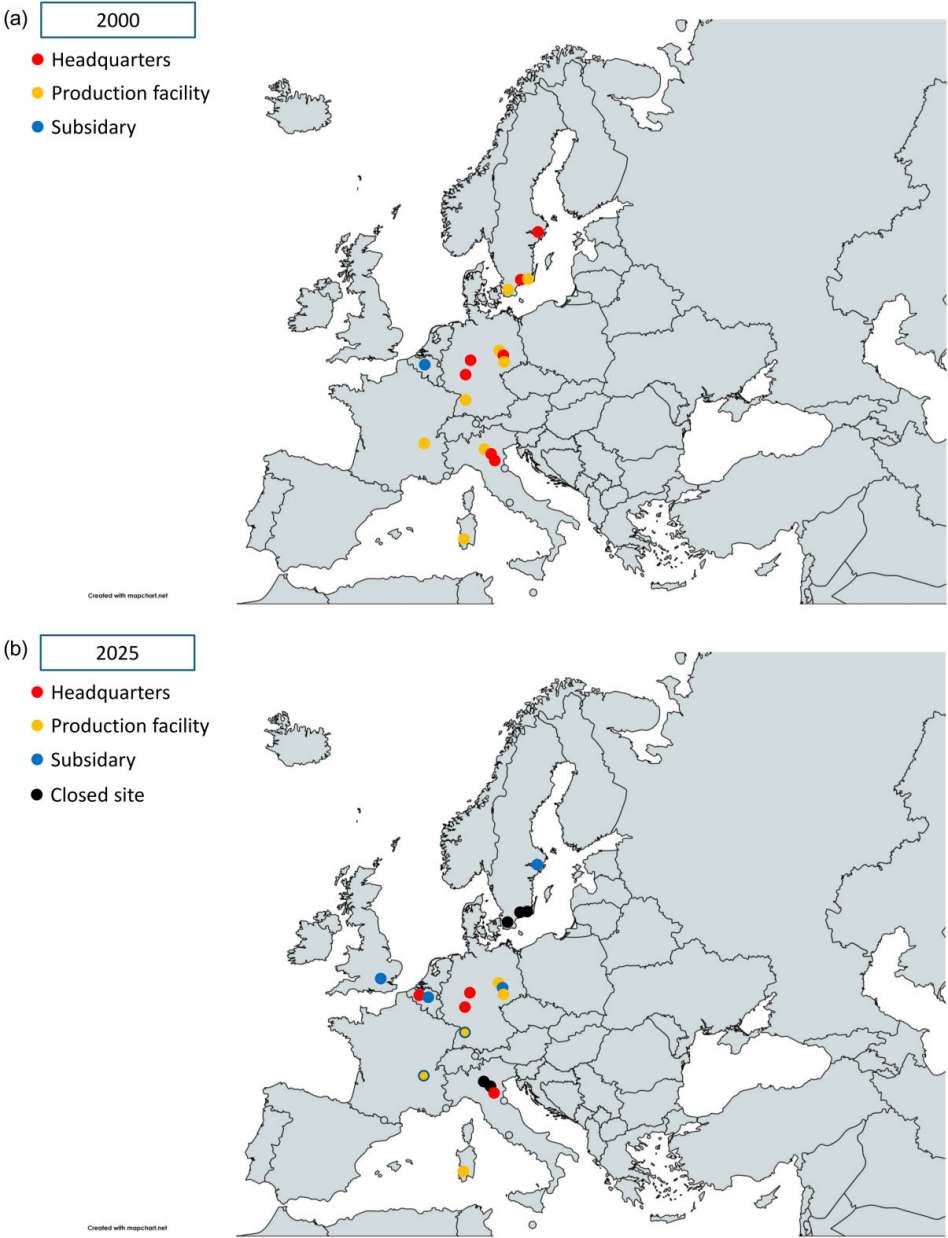
Map of Europe describing the industry base in (**a**) 2000 and (**b**) 2025. On the map, relevant locations are marked as company headquarters (red), production facilities (orange) and subsidiaries (blue). Sites that are closed between 2000 and 2025 are marked by black dots.

The dialysis industry landscape in Europe has evolved significantly over time. By the end of the 20th century, Europe was home to several headquarters of global dialysis companies that produced dialysis devices primarily in Europe (Fig. 1). However, during the last decade, mergers and acquisitions have profoundly reshaped the dialysis landscape, reducing Europe’s role. Simultaneously, globalization introduced other market players to the EU, with some companies emerging as major market innovators and others taking on manufacturer and/or supplier roles. Overall, the noticeable changes in the EU dialysis market are characterized by less innovation and increased commoditization. The current landscape remains fluid, with the recent entry of private equity into several key market players leading to changes in the distribution of HD devices. For instance, one major private equity owned dialysis company has mostly withdrawn its chronic HD portfolio from the Western European market, although (at least for now) it still produces dialysers in two plants in Germany and France. Despite these market dynamics, several market trends are worth noting.

Currently, there is an abundance of ‘haemo(dia)filters’. A recent study identified more than 300 different devices from several suppliers across the globe. There is a wide range of offerings with a broad range of relevant characteristics, i.e. a range of membrane surface areas in graded size ranges for children and adults, filters with high dialyser mass transfer-area coefficient [23], both low and high membrane ultrafiltration coefficients (DK_UF_), a range of sieving coefficients for different solutes, different polymers, several surface-treated dialysers (e.g. heparin-coated, vitamin E–coated) and different sterilization methods. In general, with respect to dialysers, the European dialysis device market encompasses a suitable offering for the needs of the vast majority of patients.

**Figure 2:**
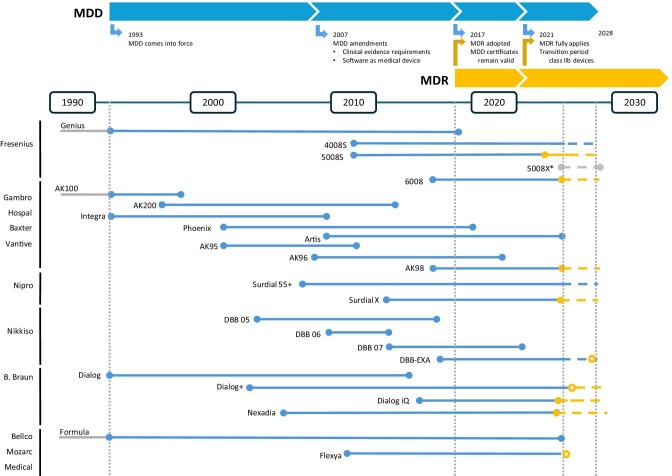
Timeline from the introduction of the biomedical device directive (MDD) to the biomedical device regulation (MDR). For different suppliers of dialysis monitors that have (had) a large market share, we provide the commercialization timeline of individual types of dialysis monitors, with type of certification (MDD—blue; MDR—orange). Closed circles indicate start and end of commercialization. Devices that are MDD certified but for which MDR recertification is planned/pending are marked with an open orange circle.

In sharp contrast, the market for ‘dialysis monitors’ has undergone significant changes. Dialysis monitors, also referred to as dialysis machines, are medical devices that deliver and regulate the treatment process to ensure patient safety and therapeutic effectiveness. Since the adoption of the MDR, marketing and sales of several dialysis monitors which previously had a significant market share has halted. As of September 2025 (8 years after the adoption of the MDR), only a small number of dialysis monitors achieved recertification under the MDR. While recertification of several devices is still pending (and is permitted by MDR until 2028), production of other devices has been halted altogether. Direct communication between co-authors and industry representatives has indicated that most companies have directed substantial research and development (R&D) resources towards recertification processes, rather than using these resources to promote true innovation. Even more telling is that 8 years after the adoption of MDR, not a single completely novel dialysis monitor for in-centre HD has received MDR certification and reached a significant market share. While the market currently offers a range of dialysis monitors for adult patients, their number is clearly reduced, and the pace of innovation has markedly slowed (Fig. 2).

The market for ‘specialized devices’ for extracorporeal circulation shows a similar picture. Dedicated niche devices are now in short supply. Specifically, haemoperfusion cartridges which were used in specific indications, e.g. charcoal haemoperfusion cartridges for certain types of intoxications, have become difficult to source, as several traditional suppliers have stopped marketing and sales in the EU. Meanwhile, new market entries are appearing, mainly from China.

One specific area confronted by a critical shortage of suitable devices and materials is ‘paediatric dialysis’ [24]. In paediatric dialysis, there is an acute lack of suitable dialysis machines and catheters to safely perform life-saving long-term HD and PD treatment in young children [25]. Paediatric nephrologists are increasingly forced to treat children with off-label dialysis machines and catheters, significantly increasing risks to the child and making the dialysis therapy suboptimal. Product specifications and regulatory issues such as the labelling of dialysis devices were obtained from all the major dialysis system manufacturers [24, 26]. None of the machines currently available is labelled for children under 10 kg body weight and many techniques to improve dialysis treatment and long-term outcomes have not been adapted for children below 40 kg [26]. The essential technical requirements needed to safely perform HD in small children are scarce—dialysis machines and catheters require specific design adapted to the needs of small children and cannot simply be miniaturized versions of adult devices. Similar shortages have developed regarding PD equipment.

Paediatric dialysis requires purpose-built, miniaturized extracorporeal devices that deliver precise control of blood flow, ultrafiltration and electrolyte balance to ensure safe and effective treatment in infants and small children. The reported ultrafiltration precision of many dialysis machines ranges widely (approximately 20–50 mL/h), a level of error that can produce clinically significant hypo- or hypervolaemia in small children. Such ultrafiltration imprecision may precipitate rapid intravascular volume shifts with intradialytic hypotension, end-organ ischaemia and cerebrovascular events [27]. Secondly, currently available dialysers and HD circuits are designed for adult physiology and remain disproportionately large for patients weighing <10 kg; thus, to maintain haemodynamic stability, blood priming is routinely required at the initiation of each session. Repeated exposure to blood from multiple donors (commonly four to five sessions per week for several months) increases the risk of transfusion-related adverse events, including blood-borne infections and alloimmunization which can reduce options for future kidney transplantation [28, 29]. Development of scaled-down dialysis systems with tightly regulated ultrafiltration control is imperative to reduce harm and improve outcomes in the paediatric population.

Thirdly, haemodiafiltration (HDF) improves survival in adult cohorts [19] and has been reported to attenuate cardiovascular damage and to improve growth and quality of life in children on maintenance HDF [30]. Currently, there are no dialysis devices that permit HDF in children weighing <10 kg, and only one dialysis machine that allows HDF in children between 10 and 15 kg.

Additional features currently unavailable for small children include real‑time intradialytic monitoring of solute clearance (e.g. urea), relative blood volume and dialysate/blood temperature permits dynamic assessment of delivered dialysis dose and allows titration of ultrafiltration, thereby improving session tolerability [31–33]. Vascular access performance can be quantified by access recirculation measurements. Although most dialysis machines incorporate these modalities, the respective software adaptations are not available for children: the Baxter AK98 lacks such monitoring options, and the Fresenius 6008 requires a minimum blood flow of ∼250 mL/min for reliable recirculation and clearance assessment—a threshold frequently unattainable in smaller children—thereby restricting the advantages of these technologies to older/larger paediatric patients. Furthermore, the automated ultrafiltration and temperature control algorithms of the 6008 are not validated for use in children <40 kg. Contemporary sodium balance control during dialysis, which may further individualize fluid and haemodynamic management [34], is available on the 6008 but currently recommended only for patients >40 kg. Collectively, these device-dependent limitations underscore the need for paediatric-specific functionality and validation to extend advanced intradialytic monitoring and therapy optimization to smaller children.

Although the knowledge and technology exist for design and manufacture of the equipment required for safe high-quality dialysis in small children, the prohibitive costs of regulations on device manufacturing is disincentivizing manufacture of devices that are only sold in small quantities.

## IMPACT OF MDR ON THE v DIALYSIS LANDSCAPE

Key point:

- The MDR has increased regulatory burdens, strained R&D, and reduced European production capacity, thereby limiting innovation and threatening the supply and availability of dialysis devices.

It is difficult to distinguish the effects of the MDR from other emerging macrotrends in the EU, amongst which are the high labor costs and steadily increasing energy and material costs, compared with other regions in the world. Remaining competitive in an open market economy has become more challenging. Several important issues can be discerned that relate to the MDR, which is hindering manufacture, supply and availability of these crucial supplies for dialysis to the EU population, and these may need to be reconsidered.

First, the MDR increases strain on R&D budgets. MDD-approved biomedical devices are still allowed to be marketed and distributed (up until 2028), with the caveat that many HD filters are produced outside Europe, the situation becomes more challenging for complex biomedical devices, e.g. dialysis monitors, which typically undergo several evolutionary cycles. Recertification under the MDR requires significantly more documentation, which consumes significant R&D resources. In specification of what was stated above, informal contact with several suppliers has indicated that R&D efforts for (re)certification are estimated to consume at least twice the resources needed for MDD and in some cases even more than 50% of overall R&D costs. In the 8 years since its adoption and four years since full implementation, it remains unclear whether and to what extent the transition from MDD to MDR has resulted in safer and better treatments. It is important to consider the associated opportunity costs; that is, the implicit cost incurred by missing out on one or more innovations due to allocation of R&D costs towards recertification processes.

Second, MDR requirements create an uneven playing field for players from different regions of the world. European companies are required to have all products, whether existing or novel, undergo MDR certification at a significant cost, while external market players can select which of their product portfolio is poised for MDR certification, based on *a priori* likelihood of success in the EU market. This change disincentivizes early-stage development in Europe as regulatory aspects are too costly and a major set-back for clinical innovation.

Third, due to inadequate capacity at the Notified Bodies responsible for device approval, delays have arisen. The EU has recognized the insufficient capacity of Notified Bodies and, thus, extended the timeframe within which manufacturers need to achieve reapproval for legacy devices. Evidently, this impacts all manufacturers operating in Europe.

Finally, we already observe a clear decline in the number of European production facilities of dialysis consumables. Dialysis manufacturers operating in Europe are significantly impacted by the stricter EU regulations and other economic factors, which renders the European market less attractive for investors. Several large manufacturing plants have closed in recent years, while others were acquired by private equity. Similar to the critical shortage of biomedical supplies seen during the COVID pandemic, these circumstances pose a risk for supply chain shortages in case of future major emergencies, such as another pandemic, or other natural or man-made disasters, e.g. the 2012 earthquake disrupting the Mirandola (Italy) biomedical cluster.

## FROM IMPASSE TO IMPACT

Key points:

- Regulatory streamlining: Regulation must protect patients without delaying access to essential dialysis devices.
- Support for innovation: Innovation in dialysis requires incentives and disease-specific expertise, particularly for vulnerable groups.
- Market and supply chain: A competitive but resilient market is vital for secure access to dialysis care.
- Research and knowledge expansion: Sustained investment in kidney research is needed to close Europe’s innovation gap.
- Policy and strategic alignment: Biomedical regulation must align safety with economic competitiveness and innovation.
- Patient-centered care: Patients and clinicians should guide regulatory and innovation priorities.

The first 50 years of the development of dialysis were characterized by open international scientific exchange, close collaboration between clinician scientists and a diverse landscape of innovative biomedical companies. Strict regulations and shifting geo-economics have had a major detrimental impact on this once-fertile environment. Now is the time to develop a new strategic, EU-wide vision for dialysis. To truly stimulate the development and approval of novel and innovative medical devices for dialysis, we must adopt a paradigm shift, which requires several necessary steps. The EU should review and develop a coherent biomedical devices strategy that integrates regulatory aspects, a competitive economic strategy with targeted incentives for scientific development and innovation.

We need a critical appraisal and modification of the MDR to address some of its shortcomings and unintended side-effects (Table 2). A core set of recommendations (Table 3) has been advanced by the biomedical alliance [35]. The regulatory efficiency should be enhanced by streamlining certification processes. The capacity of notified bodies needs to be increased. This should be accompanied by the establishment of a new coordinating division at the European Medicines Agency. In addition, support for innovation should be strengthened to foster the development of new medical devices. The significant societal impact of care for patients with kidney failure, with high financial costs for both patients and society, would benefit from targeted EU scientific resources for innovation in biomedical devices and their clinical application.

**Table 1:** Biomedical devices in kidney failure and the impact of the MDR.

Type of device	R&D costs	Time to certification	Device availability
Dialysis filter	Moderate	Moderate	Good
Dialysis monitor	High	Long	At risk
Paediatric devices	High	Long	Critical
Specialty adsorbers	Moderate	Long	Poor to critical

The MDR was adopted in 2017 and became fully applicable in 2021. Effects of the MDR are expressed using a semi-quantitative scale. Colour codes represent degree of impact with green no notable impact, orange moderate impact and red indicating severe impact. Data reflect the status at 1 September 2025.

**Table 2:** Key threats to patient care and medical innovation.

• Excessive certification costs creating financial burdens for manufacturers
• Insufficient clinical use data on specific medical devices
• Lack of transparency in regulatory processes
• Limited access to orphan and paediatric devices, jeopardizing patient care
• Increasing risk of devices being withdrawn from the EU market due to regulatory complexity
• Barriers to innovation that stifle research and development
• Delays in the implementation of new medical devices
• Increasing regulatory complexity adding uncertainty to the system
• Fragmented governance leading to inconsistencies in implementation across Member States
• Dependence on imports from distant (predominantly southeast Asian) countries augmenting supply chain vulnerabilities

**Table 3:** Action points for EU policymakers and regulators.

**Generic points—developed by biomedical alliance**
• Enhancing regulatory efficiency by streamlining certification processes and increasing the capacity of notified bodies
• Establishing a new coordinating division at the European Medicines Agency
• Ensuring patient access to critical medical technologies by improving market availability
• Strengthening support for innovation to foster the development of new medical devices
• Increasing transparency and stakeholder engagement to create a more predictable regulatory environment
**Dialysis specific action points**
• Regulatory streamlining: regulation must protect patients without delaying access to essential dialysis devices
• Support for innovation: innovation in dialysis requires incentives and disease-specific expertise, particularly for vulnerable groups
• Market and supply chain: a competitive but resilient market is vital for secure access to dialysis care
• Research and knowledge expansion: sustained investment in kidney research is needed to close Europe’s innovation gap
• Policy and strategic alignment: biomedical regulation must align safety with economic competitiveness and innovation
• Patient-centred care: patients and clinicians should guide regulatory and innovation priorities
• Integrating the breadth of dialysis needs (including children) into emergency preparedness plans

Part of this strategy should be focused on niche biomedical devices (such as paediatric dialysis devices and specialty adsorbers), for which the current market does not provide the necessary life-saving devices. One way forward would be to adopt a strategy akin to that successfully implemented by the EU for paediatric and orphan drugs. The development of paediatric-specific medications was spurred by regulations that provided financial incentives such as patent extensions, and mandatory paediatric investigation plans that ensured both economic viability and rapid innovation. We propose that a parallel approach be adopted for medical devices: implementing targeted incentives, including legal adaptations and financial benefits, encouraging industry to develop safe, effective and CE-labelled dialysis devices. Furthermore, integrating paediatric disease-specific expertise into existing MD evaluation processes is crucial. These expert groups should perform pre-evaluations and advocate for timely approval processes. Clear representation of paediatric considerations within existing evaluation authorities will facilitate innovation, ensuring that the most critical needs of children are not sidelined by regulatory or economic hurdles.

While amendment of the MDR is necessary, its effectiveness hinges on the integration with other regulations, as well as economic considerations. As physicians we believe that an open market for biomedical devices, where true competition is not unnecessarily hindered by unilateral regulations, best serves the needs of our patients, permitting availability of a wide array of innovative treatments from across the globe. An open market, however, does not necessarily equate cost-driven commoditization. As physicians working in Europe we are ill-positioned to judge market dynamics outside of Europe. While preparing this document we learned that from 19 June 2025, the European Commission adopted the first measure under the International Procurement Instrument, introducing targeted restrictions on access to the EU public procurement market for medical devices originating from the People’s Republic of China. This regulation excludes companies from the People’s Republic of China from participating in EU public tenders for medical devices above €5 million and limits the share of medical devices originating from the People’s Republic of China to no more than 50% in relevant contracts. The measure is designed to incentivize the People’s Republic of China to open its public procurement market by removing the persistent barriers that EU suppliers face when attempting to access it. Currently, it is unclear whether this includes public tendering of dialysis devices in individual EU member states.

Another critical aspect for which clear policy-making is required is supply chain resilience. Given that KRT is lifesaving, even short-term disruption will lead to avoidable morbidity and mortality [36]. It is up to the EU to provide regulations and/or incentives to ensure resilience to supply chain disruptions. It is up to industry to come up with potential solutions, including geographic spread of manufacturing sites that permit local-for-local production, as well as maintaining strategic stocks of essential products. The European kidney community should develop scenario-based requirements to support disaster-based plans and policies, as described in the American Society of Nephrology’s dedicated website (https://epc.asn-online.org/projects/epr/).

Finally, we recommend a clear EU-wide strategy for research and innovation in kidney failure. According to research for the European Commission into so-called ‘high-burden under-researched medical conditions’, CKD research is one of the most underfunded fields of medicine [37]. One potential model could be the foundation of an EU-funded institute for chronic diseases including kidney disease, akin to the US-based National Institute of Diabetes, Digestive and Kidney Diseases, part of the National Institutes of Health.

## CONCLUSION

In conclusion, the EU MDR, while aimed at enhancing the safety of medical devices, has inadvertently created barriers that threaten the availability and innovation of vital dialysis equipment. In recent years, nephrologists have faced difficulties in obtaining supplies of certain biomedical devices or drugs, while patients’ lives depend on the availability of these products. Increased regulatory burdens have led to the withdrawal of a substantial number of essential dialysis devices, leaving clinicians with increasingly limited options and risking worsening patient outcomes. The regulation’s rigidity hampers innovation, especially for devices required in rare disease populations such as children. The current situation demands urgent action from the EU to mitigate against a shortage of dialysis equipment, to modernize regulation with targeted incentives, and as a first step towards this, to establish specialized, disease-focused expert panels within existing medical device evaluation groups. This requires bringing together the joint expertise from patients, caregivers, healthcare professionals, technique experts, health economists and industry leaders. For people with kidney disease, their lives depend on it, and it is our moral and professional obligation to prioritize their health and their future.

## Data Availability

The data underlying this article will be shared on reasonable request to the corresponding author.
